# Oral Prevention and Management of Oral Healthcare

**DOI:** 10.3390/ijerph18041970

**Published:** 2021-02-18

**Authors:** Iole Vozza

**Affiliations:** Department of Oral and Maxillo-Facial Sciences, Sapienza University of Rome, via Caserta, 6-00161 Rome, Italy; iole.vozza@uniroma1.it

Oral health is an important factor in the maintenance of general health, wellbeing, and overall quality of life. Envisioning a transformational change in the management of oral healthcare, this Special Issue is focused on oral health and prevention and their impact on clinical practice current oral health literacy and policies.

Preventive strategies should be implemented to reduce oral problems, due to the negative consequences on individuals and communities in terms of pain and suffering, functional impairments, and reduced quality of life.

The Special Issue demonstrated that the national guidelines for the prevention and clinical management of traumatic dental injuries in developmental age published by the Italian Ministry of Health are not uniformly applied [1]. Covello F showed that oral piercings can represent a risk to oral health and that there is a widespread lack of awareness regarding the complications and correct methods of maintaining oral piercings. Periodic checks by both dentists and dental hygienists for patients with oral piercings could play a decisive role in preventing, intercepting and treating the complications that they can cause [2]. Early diagnosis of occlusal caries is of paramount importance for a minimally invasive approach in dentistry. The Special Issue showed that new technologies are fundamental in order to avoid underestimation [3]. New technologies for oral healthcare sustainability are clearly described by Palaia G [4] through use of the 445 nm diode laser, which proved to be a device that can be safely used for the biopsies of clinically unsuspicious lesions. Sfasciotti GL [5] clarified that, in pediatric patients with a high labial frenulum attachment, a diode laser device is more suitable, compared to the CO_2_ device, in avoiding further recession. Meanwhile, Impellizzeri confirmed that photobiomodulation therapy [PBMT] can accelerate orthodontic movements, due to its biostimulating and regenerative capacity on soft tissues, as a consequence of the increase in differentiation, proliferation, and activity of cells that are involved with alveolar bone remodeling [6]. Cavalcante NV [7] illustrated that NutriOdonto software can contribute to the decision making of managers and professionals and to improving actions in health promotion and oral disease prevention.

Psychosocial factors, such as improved awareness, knowledge, and attitudes toward dental health care in both children and adult patients, both healthy or special needs, are included in general health status improvements.

Di Carlo G [8] showed the relevance of sleep disorders in the pediatric population and highlighted the central role of pediatric dentists in the earlier diagnosis of these disorders. Oku S [9] found low oral function in university students and suggested its association to low OHRQoL in this population. Schulz-Weidner N [10] demonstrates that parents of children with congenital heart disease [CHD] underestimate the importance of improving oral health. The low priority for a good oral hygiene and a healthy diet can be a risk factor for odontogenic bacteremia and infective endocarditis. Paszynska E [11] proved the evaluation of the association between biomarkers of early primary arterial hypertension [HA] and oral diseases among children and adolescents. This may indicate future strategies for preventive measures for hypertensive children and adolescents. Zhurakivska K [12] focused on malignant fibrous histiocytoma as one of the most common soft tissue sarcomas in adults and found that further studies are necessary in order to standardize the approach to patients affected by oral malignant fibrous histiocytoma.

This Special Issue also focused on childhood and adolescence, finding that the primary oral disease found during this period is dental caries. Tudoroniu C [13] demonstrated that dietary habits and irregular dental check-ups were associated with the occurrence of dental conditions. Davidovich E [14] shows that children’s oral health is influenced by toothbrush type, starting age of brushing, compliance with twice-daily brushing, and bite abnormalities. Štefanová E [15] found that the absence of daily toothbrushing was associated with a lower socioeconomic situation and so the improvement of oral hygiene in lower socioeconomic populations is needed. In fact, Jeon, J.-E [16] showed that implementing oral health programs can decrease social inequality with a relative intensification in use of dental sealants in vulnerable people.

In implantology, dental implants are one of the most commonly used ways to replace missing teeth. Nevertheless, the ensuing close contact with hard and soft oral tissues exposes these devices to infectious peri-implant diseases. To prevent such infection, several surface treatments have been developed in the last few years to improve the antimicrobial properties of titanium dental implants. Pranno N [17] found, in an in vitro pilot study, that the surface treatment of titanium disks with graphene nanoplatelets represents a promising solution to improve the antibacterial activity of titanium implants. The surgical treatment of peri-implantitis is currently based on the removal of biofilms from the implant surface by primary means of mechanical and physical treatments. Lollobrigida M [18] shows that such approaches often determine some implant surface alterations with detrimental effects on re-osseointegration and as air powder abrasion with glycine powder, and 3 W diode laser had the lowest impact on surface physicochemical properties. In order to evaluate oral status, reasons for tooth extractions and related risk factors from the study of Passarelli PC [19], it has emerged that caries and periodontal disease were the most common causes of extraction in the adult population; therefore, screening strategies might be required for the early interception of caries and periodontal disease and decrease the number of tooth extraction. Knowledge of prevention is also important among teachers because schools are the second houses of children and, from the study of Uzarevic Z [20], it was found that future primary school teachers have a lack of knowledge for immediate reply to tooth avulsion, leaving poor chances for a successful prognosis of tooth replantation.

Finally, regarding oral prevention, it is impossible to forget oral precancerosis. There is a statistically significant correlation between tobacco smoking and the presence of oral leukoplakia. Early diagnosis and implementation of appropriate treatment of leukoplakia, elimination of the main risk factor, which is smoking, and the improvement of effective tobacco control interventions are very important [21].

I hope that the readers of the *International Journal of Environmental Research and Public Health* will find these multi-, inter-, and transdisciplinary perspectives about oral prevention and the management of oral healthcare both informative and inspiring.
